# During infection of epithelial cells *Salmonella enterica* serovar Typhimurium undergoes a time-dependent transcriptional adaptation that results in simultaneous expression of three type 3 secretion systems

**DOI:** 10.1111/j.1462-5822.2007.01099.x

**Published:** 2008-04-01

**Authors:** I Hautefort, A Thompson, S Eriksson-Ygberg, M L Parker, S Lucchini, V Danino, R J M Bongaerts, N Ahmad, M Rhen, J C D Hinton

**Affiliations:** 1 Molecular Microbiology Group, Institute of Food Research Norwich NR4 7UA, UK; 2 Department of Microbiology, Tumor and Cell Biology, Karolinska, Institute, Nobels väg 16 Stockholm 171 77, Sweden; 3 Imaging Partnership, Institute of Food Research Norwich NR4 7UA, UK

## Abstract

The biogenesis of the *Salmonella*-containing vacuole within mammalian cells has been intensively studied over recent years. However, the ability of *Salmonella* to sense and adapt to the intracellular environment of different types of host cells has received much less attention. To address this issue, we report the transcriptome of *Salmonella enterica* serovar Typhimurium SL1344 within epithelial cells and show comparisons with *Salmonella* gene expression inside macrophages. We report that *S.* Typhimurium expresses a characteristic intracellular transcriptomic signature in response to the environments it encounters within different cell types. The signature involves the upregulation of the *mgtBC*, *pstACS* and *iro* genes for magnesium, phosphate and iron uptake, and *Salmonella* pathogenicity island 2 (SPI2). Surprisingly, in addition to SPI2, the invasion-associated SPI1 pathogenicity island and the genes involved in flagellar biosynthesis were expressed inside epithelial cells at later stages of the infection, while they were constantly downregulated in macrophage-like cells. To our knowledge, this is the first report of the simultaneous transcription of all three Type Three Secretion Systems (T3SS) within an intracellular *Salmonella* population. We discovered that *S.* Typhimurium strain SL1344 was strongly cytotoxic to epithelial cells after 6 h of infection and hypothesize that the time-dependent changes in *Salmonella* gene expression within epithelial cells reflects the bacterial response to host cells that have been injured by the infection process.

## Introduction

In humans and many animals, *Salmonella enterica* causes a multistage systemic infection that involves invasion and crossing of the epithelial cell barrier and subsequent intracellular replication in monocytic cells. Regarding *S. enterica*, and the related enteric pathogens *Shigella* and *Yersinia*, much of the infection-associated cross-talk with host cells is directed by bacterial type III secretion systems (T3SSs) (Hansen-Wester and Hensel, 2001; Ghosh, 2004). T3SSs enable bacteria to translocate virulence proteins, also called effector proteins, from the bacteria into the host cell often to specifically interfere with host cell functions such as actin polymerization, signal transduction and apoptosis (Hansen-Wester and Hensel, 2001). *S. enterica* possesses two classical T3SSs encoded by *Salmonella* pathogenicity island 1 (SPI1) and *Salmonella* pathogenicity island 2 (SPI2) (Hansen-Wester and Hensel, 2001). In addition, *Salmonella* possesses a third T3SS responsible for the flagellar-based motility of the pathogen (Macnab, 2004; McCarter, 2006).

SPI1 plays a fundamental role in the early stages of mammalian infection through triggering Cdc42- and Rac1-mediated remodelling of the actin cytoskeleton of the host cell, and leading to internalization of the bacteria and subsequent penetration of the ileal mucosal lining (Galan and Curtiss, 1989; Hansen-Wester and Hensel, 2001). SPI1 also has a pro-inflammatory potential through its ability to activate JNK- and p38-dependent nuclear responses (Hobbie *et al*., 1997) and the release of IL-1β (Monack *et al*., 2001). Furthermore, selected SPI1-associated effector proteins cause a very strong pro-apoptotic effect in monocytic cells (Hersh *et al*., 1999). Concomitantly, SPI1-deficient mutants show a 10-fold reduction of LD_50_ in mice when given orally, whereas such mutants retain virulence via the intraperitoneal route, illustrating the importance of SPI1 in the early stages of infection, such as the invasion process (Bajaj *et al*., 1996).

Once intracellular in the endosomal compartment, *S.* Typhimurium takes control of the trafficking and evolution of the internalized vacuole, to form the *Salmonella*-containing vacuole (SCV). The SCV interacts with the early endocytic pathway but resists fusion with lysosomes in both non-phagocytic and phagocytic cells, enabling *Salmonella* to avoid host cell defences (Harrison *et al*., 2004; Marsman *et al*., 2004). Intracellular survival and systemic spread of *Salmonella* in the murine typhoid infection model relies on SPI2 and SPI2-associated effector proteins and their ability to interfere with vesicular trafficking of the host cell. SPI2 functions to protect the SCV from the effect of the phagocytic defence enzymes, such as phagocyte NADPH oxidase and inducible nitric oxide synthase (Mastroeni *et al*., 2000; Vazquez-Torres *et al*., 2000; Chakravortty *et al*., 2002). In addition, selected SPI2-associated effector proteins allow *S.* Typhimurium to polymerize actin in the vicinity of the SCV (Meresse *et al*., 2001; Poh *et al*., 2007). *S.* Typhimurium effectors also cause accumulation of microtubules around the SCV (Kuhle *et al*., 2004), acquisition of several endosomal markers (Steele-Mortimer *et al*., 1999) and recruitment of kinesin and dynein to regulate vacuolar membrane dynamics (Guignot *et al*., 2004; Marsman *et al*., 2004; Boucrot *et al*., 2005). Certain SPI2 effector functions are also associated with a slow-progressing induction of apoptosis in monocytic cells (Monack *et al*., 2001), and interference with the activity of the ubiquitination pathway in professional phagocytes (Rytkonen *et al*., 2007).

Whether or not the flagellar T3SS plays a distinct role in *S.* Typhimurium infection remains controversial. It is agreed that the flagella and motility system is generally associated with the extracellular life of the pathogen and that motility can assist invasion. The flagellar T3SS also possess a pro-inflammatory potential through its interaction with toll-like receptor (TLR) 5 (Reed *et al*., 2002; Choudhary *et al*., 2005) or with the cytosolic nod-like receptors (NLR), such Birc 1e and Ipaf (Franchi *et al*., 2006; Miao *et al*., 2006; Molofsky *et al*., 2006; Coers *et al*., 2007). The TLR-mediated recognition of flagellin triggers induction of a pro-inflammatory cascade, including IL-8 secretion that will recruit phagocytic cells such as neutrophiles to the site of infection (Ciacci-Woolwine *et al*., 1998; Eaves-Pyles *et al*., 2001; Gewirtz *et al*., 2001a,b; Hayashi *et al*., 2001; Zeng *et al*., 2006). The NLR-mediated cytosolic recognition of flagellin results in caspase 1-dependent IL-1β activation, leading to host cell death and a T cell-mediated immune response (Inohara *et al*., 2005).

While it is clear that internalization and subsequent intracellular replication of *S. enterica* are central T3SS-directed events of a systemic infection, these activities are not sufficient on their own to promote pathogenesis. The successful infection of a host by *Salmonella* requires a delicate interplay of several metabolic functions, including the ability to synthesize aromatic amino acids and nucleotides. Additionally, the capacity to express virulence functions must be integrated and controlled by the general gene regulatory programs that steers the responses and metabolic activities of the bacterial cell (Rhen and Dorman, 2005). For example, *aroA* or *purE* mutants retain their ability to invade mammalian cells but are unable to subsequently replicate in mice (Hoiseth and Stocker, 1981; Fields *et al*., 1986), whereas mutants defective in the conserved *ompR/envZ* or *phoP/phoQ* regulatory pathways remain attenuated in both cultured cells and in mice (Groisman, 2001). Although various nutritional and environmental signals necessary for the adaptation and survival of *Salmonella* have been investigated for decades, the environmental differences between host cell types and their impact upon *Salmonella* gene expression remain to be understood.

*S*. Typhimurium targets both epithelial cells and macrophages during infection of the gastrointestinal tract (Frost *et al*., 1997; Tsolis *et al*., 1999), but it is apparent that the two types of cells differ in their response to infection. For example, the timing of acquisition and exclusion of endosomal markers differs between macrophages and epithelial cells (Knodler and Steele-Mortimer, 2003); non-phagocytic cells also support different levels of *S.* Typhimurium intracellular replication, which begins at 3–4 h post infection (p.i.) in epithelial cells but is delayed until 4–8 h p.i. in macrophages (Gahring *et al*., 1990; Knodler and Steele-Mortimer, 2003; Eriksson-Ygberg *et al*., 2006). Initiation of the formation of *Salmonella*-induced filaments (Sifs) occurs at 5–6 h p.i. inside epithelial cells but not until 9 h p.i. inside macrophages (Garcia-del Portillo *et al*., 1993; Garcia-del Portillo and Finlay, 1995; Stein *et al*., 1996; Knodler *et al*., 2003). Finally, the maintenance of the SPI2-mediated condensation of actin appears to be essential for bacterial replication inside macrophage-like cells, but not for bacterial replication within epithelial cells (Meresse *et al*., 2001).

The recent ability to profile bacterial gene expression inside host cells (Eriksson *et al*., 2003; Hinton *et al*., 2004) has brought new insight into the infection biology of pathogens such as *Listeria monocytogenes*, *S. enterica, Sh. flexneri* and *Mycobacterium tuberculosis* (Eriksson *et al*., 2003; Schnappinger *et al*., 2003; Chatterjee *et al*., 2006; Lucchini *et al*., 2006). Furthermore, a combined approach using microarray and selective capture of combined sequences identified genes of *S. enterica* serovar Typhi that responded to the macrophage SCV environment (Faucher *et al*., 2006).

We describe here the transcriptomic analysis of *S.* Typhimurium SL1344 inside epithelial cells, and focus on similarities and differences to the bacterial gene expression profile inside macrophage-like cells reported by Eriksson *et al*. (2003). The HeLa epithelial cell line was chosen because it is a well-defined model for infection of mammalian cells with *S.* Typhimurium, and has been used to characterize the biogenesis and evolution of the SCV (Steele-Mortimer *et al*., 1999; Beuzon *et al*., 2000). Additionally, we identify novel time-dependent changes in *Salmonella* gene expression that occur inside epithelial cells, revealing simultaneous expression of the three *Salmonella* T3SS. We present the first evidence that *S.* Typhimurium not only alters its gene expression profile according to cell type, but also shows large transcriptomic changes that reflect alterations in host cell biology.

## Results and discussion

HeLa cells have commonly been used to model infection of epithelial cells by *S.* Typhimurium and various other bacterial pathogens (Walz *et al*., 1985; Stibitz *et al*., 1988; Rosqvist *et al*., 1990; Garcia-del Portillo and Finlay, 1995; Wang *et al*., 1996; Boucrot *et al*., 2003; Jabado *et al*., 2003; Unsworth *et al*., 2004; Way *et al*., 2004; Birmingham *et al*., 2005; Lucchini *et al*., 2005). In our study, we have determined the gene expression profile of *S.* Typhimurium inside HeLa cells at 2, 4 and 6 h p.i., as these time points coincide with distinct stages of the intracellular infection process. At 2 h p.i., *S.* Typhimurium is inside a vacuolar compartment but has not yet begun to replicate intracellularly (Gorvel and Meresse, 2001; Steele-Mortimer *et al*., 1998). The 4 and 6 h time points reflect stages of infection when *S.* Typhimurium has begun to grow inside epithelial cells (Knodler and Steele-Mortimer, 2003).

**Fig. 1:**
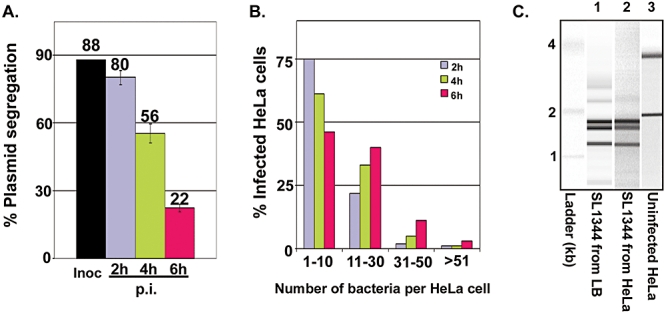
Intracellular replication and distribution of *S*. Typhimurium SL1344 *Salmonella* strain inside epithelial cells. A. Bacterial replication was measured by following the segregation of the pPIR plasmid, which only replicates at 30°C. During each bacterial division at 37°C, only one daughter cell inherits the pPIR plasmid. Plasmid segregation is expressed as the percentage of bacterial cells that retain pPIR. The difference in percentage of bacterial cells carrying the pPIR plasmid inside epithelial cells between 2 and 6 h p.i. reflects a fourfold increase in the population level. Inoculum (Black), Epithelial cells 2 h p.i. (Purple), epithelial cells 4 h (Green) and epithelial cells 6 h (Magenta). B. Distribution of *S.* Typhimurium cells inside epithelial cells at 2, 4 and 6 h p.i.; values shown are the percentage of epithelial cells containing different numbers of bacteria observed by light microscopy (*Experimental procedures*). Epithelial cells 2 h p.i. (Purple), epithelial cells 4 h p.i. (Green) and epithelial cells 6 h p.i. (Magenta). Over 150 infected HeLa cells were examined. C. Isolation of high quality bacterial RNA from infected HeLa cells and separation by size chromatography (*Experimental procedures*). Total RNA was extracted from *S.* Typhimurium grown in LB medium *in vitro* (lane 1), *S.* Typhimurium from infected epithelial cells (lane 2) and eukaryotic RNA isolated from uninfected HeLa cells (lane 3).

To validate our experimental conditions, we first confirmed that *S.* Typhimurium SL1344 was replicating inside epithelial cells by measuring the segregation rate of a temperature-sensitive plasmid (pPir) (Posfai *et al*., 1997; Clements *et al*., 2002). We observed that four times fewer bacteria harboured the plasmid at 6 h p.i. compared with 2 h p.i. (Fig. 1A), indicating that at least two generations of replication had occurred intracellularly over this period of time. Additionally, we monitored the distribution of SL1344 inside epithelial cells at 2, 4 and 6 h p.i. This analysis showed that 75% of the infected epithelial cells harboured between one and 10 bacteria at 2 h p.i. (Fig. 1B), consistent with data obtained for another *S.* Typhimurium strain 12023 (also known as 14 038) in HeLa cell infection experiments (Steele-Mortimer *et al*., 1999; Beuzon *et al*., 2002). At 2 h p.i., less than 25% of the infected epithelial cells contained more than 10 bacteria. At 6 h p.i., this number rose to more than 50% (Fig. 1B), agreeing with the level of intracellular growth determined by plasmid segregation (Fig. 1A).

We optimized the maximal recovery of bacterial cells, and prepared stabilized bacterial RNA with minimal contamination by eukaryotic RNA as described previously (Eriksson *et al*., 2003; Hinton *et al*., 2004) (Fig. 1C). We determined the transcriptome of *S.* Typhimurium SL1344 inside HeLa epithelial cells, and compared it with transcriptomic data for SL1344 within J774-A.1 macrophages (Eriksson *et al*., 2003; Lucchini *et al*., 2005). We refer to these host cell types as epithelial and macrophage cells respectively. To determine the similarities and differences between the host cell types, we used a mid-logarithmic Luria–Bertani (LB) culture of SL1344 as a common comparator, and data were statistically filtered as described in *Experimental procedures* (Table S1). We note that transcriptomic and RT-PCR data represent the average bacterial gene expression occurring in a given environment as they reflect the entire population of intracellular *Salmonella*. We nevertheless intend that this novel transcriptomic data set will provide useful insights into the process of *Salmonella* infection.

### The intraepithelial transcriptome of *S*. Typhimurium shows that many genes required for *Salmonella*-mediated enteritis and growth within epithelial cells are highly expressed

We observed that 1377 *S.* Typhimurium genes were either upregulated or downregulated by at least twofold inside epithelial cells at 2 h p.i., and 1221 genes at 6 h p.i. compared with cultures grown in LB (Table 1). These included genes such as *lpp*, *fadF* (also known as *yafH*)*, rfbA*, *sitABCD* and several SPI2-associated genes that have been shown to contribute to replication of *S.* Typhimurium in cultured epithelial cells (Watson *et al*., 1995; Spector *et al*., 1999; Maurer *et al*., 2000; Steele-Mortimer *et al*., 2002; Morgan *et al*., 2004) (Table S1).

**Table 1:** Numbers of *S.* Typhimurium genes regulated during infection of epithelial cells and macrophages.

Comparisons	Number of upregulated genes	Number of downregulated genes	Total number of differentially expressed genes
HeLa 2 h versus LB	724	653	1377
HeLa 6 h versus LB	605	616	1221
J774 4 h versus LB	938	935	1873
HeLa 6 h versus HeLa 2 h	120	67	187
J774 12 h versus J774 4 h	49	4	53
HeLa 2 h versus J774 4 h	368	259	627
HeLa 6 h versus J774 4 h	529	503	1032

Numbers of *S.* Typhimurium genes that are differentially expressed between two conditions are shown after statistical analysis and data filtering (*Experimental procedures*); the values indicated are the numbers of genes that are upregulated or downregulated by at least than twofold.

Infection of epithelial cells is a prerequisite for the induction of enteritis in the calf infection model, which has been proposed as a tool to simulate human *Salmonella*-mediated gastroenteritis (Frost *et al*., 1997). Morgan *et al*. used signature-tagged mutagenesis to identify 98 genes that were essential for calf enteritis (Frost *et al*., 1997; Tsolis *et al*., 1999; Morgan *et al*., 2004). To functionally validate our transcriptomic data, we established the relationship between our *Salmonella* gene expression data in HeLa cells and the genes previously shown to be essential for calf enteritis. This comparison (Table 2) lists the 50 *S.* Typhimurium genes that are required for bovine enteritis and that are most highly expressed in HeLa cells (Table 2 and Table S17). Our data showed that the adaptation of *Salmonella* to cultivated epithelial cells involved the expression of genes with proven roles in *Salmonella* virulence. It was apparent that genes listed that showed high levels of expression inside epithelial cells were expressed at lower levels inside macrophages (Table 2).

**Table 2:** Genes required for *Salmonella*-associated enteritis in the bovine infection model and for survival and replication in cultivated epithelial cells are expressed within HeLa cells.

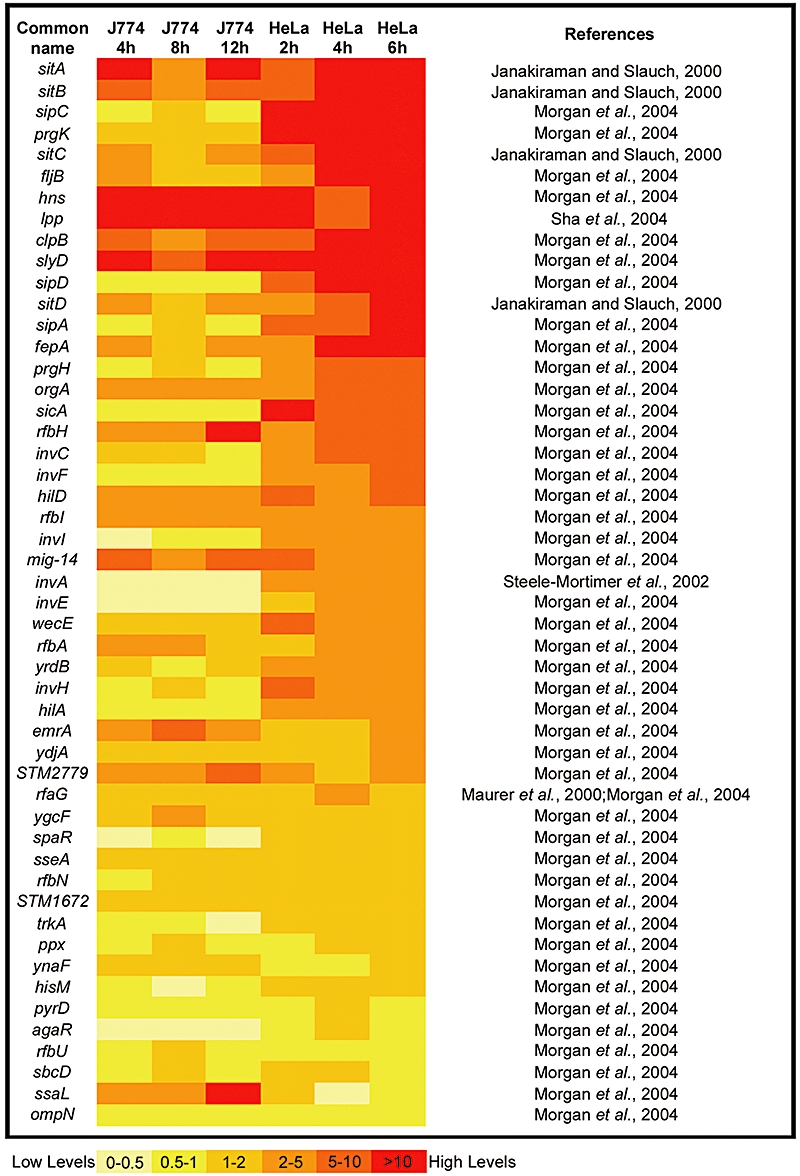

The table shows 50 *S.* Typhimurium genes that are required for replication in epithelial cells (Janakiraman and Slauch, 2000; Maurer *et al*., 2000; Steele-Mortimer *et al*., 2002) or enteritis in the bovine infection model (Morgan *et al*., 2004) and are highly expressed in HeLa cells. Each gene is colour-coded according to its level of gene expression (i.e. signal ratio of cDNA versus genomic DNA). Highly expressed genes are red,and weakly expressed genes are pale yellow. The gene list was sorted by values for *S.* Typhimurium gene expression in HeLa at 6 h p.i., and the data are taken from Table S1.

Our transcriptomic data also confirmed previous reporter gene-based analyses of *S.* Typhimurium in MDCK epithelial cells that showed upregulation of genes involved in magnesium (*mgtBC*) and phosphate transport (*pstACS*) (Garcia-del Portillo *et al*., 1992), and the virulence plasmid pSLT-associated intracellular replication gene *spvB* (Fierer *et al*., 1993). The expression of the genes involved in *de novo* biosynthesis of purines was induced up to sevenfold in epithelial cells, and the *pnuC* gene encoding a nucleoside/purine/pyrimidine transporter was twofold upregulated in epithelial cells at 6 h p.i. compared with LB (Table S1).

The transcriptomic data reported here are supported by both mutant and gene fusion-based studies that involve epithelial cells, indicating that our approach is valid for the investigation of early events of *Salmonella* infection.

### Similarities between the *S*. Typhimurium transcriptome within epithelial and macrophage cells

As the intracellular infection of epithelial cells and macrophages by *S*. Typhimurium differ in terms of kinetics and maturation of the SCV, we compared the *S.* Typhimurium SL1344 transcriptomes within HeLa epithelial cells with the data we previously obtained for *S.* Typhimurium SL1344 in macrophage-like J774-A.1 cells (Eriksson *et al*., 2003).

**Fig. 2:**
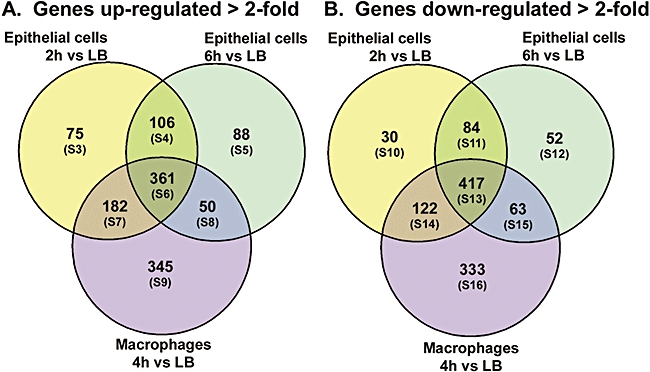
Common intracellular transcriptomic signature and cell type specific responses of *S*. Typhimurium in epithelial cells and macrophages. Non-proportional Venn diagrams are shown. A. The *S.* Typhimurium genes significantly upregulated by at least twofold from epithelial (2 h p.i.), epithelial (6 h p.i.) and macrophage cells (4 h p.i.), all in comparison with LB. B. Genes significantly downregulated by at least twofold in the same samples. The common intracellular signature of *S.* Typhimurium is represented by the central area of each Venn diagram which contains genes that show similar patterns of regulation inside both types of mammalian cells (Tables S6 and S13). The Venn diagrams were also used to define lists of genes that were only upregulated or downregulated inside macrophages (Tables S9 and S16) or only inside epithelial cells (Tables S3–S5 and S10–S12).

**Fig. 3:**
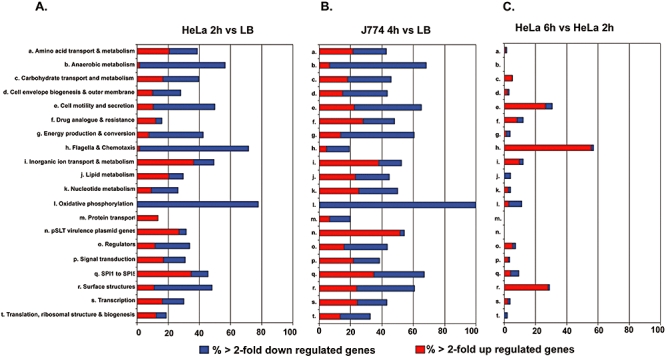
Functional categories of *S*. Typhimurium genes changing in expression in epithelial cells and macrophages. The Red and Blue bars indicate the percentage of genes of each functional category upregulated or downregulated, respectively, inside epithelial cells (2 h p.i.) versus LB (A), or inside macrophages (4 h p.i.) versus LB (B). The classes of genes differentially regulated in epithelial cells at 6 h p.i. compared with the 2 h time point are shown in (C). The numbers of genes involved in each analysis are shown in Table 1. The list of genes included in each functional category was obtained from the Kyoto Encyclopedia of Genes and Genomes, KEGG (http://www.genome.jp/kegg/).

**Fig. 4:**
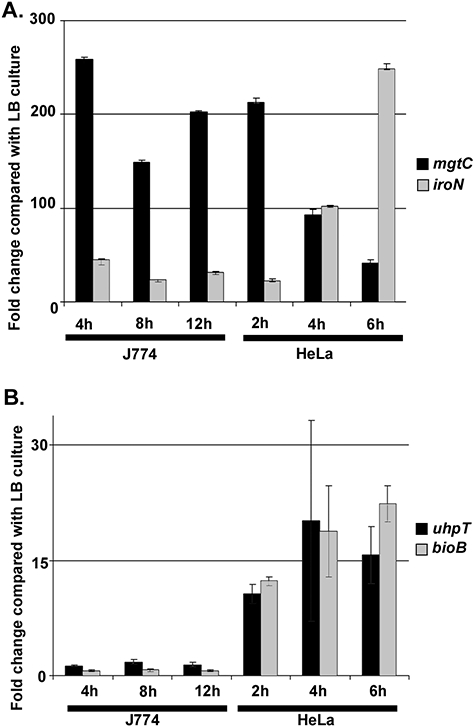
Selected *S*. Typhimurium genes that show differential expression between epithelial cells and macrophages. Transcriptomic data are shown for the *S.* Typhimurium *mgtC* and *iroN* genes (A), *uhpT* and *bioB* genes (B) inside both macrophage and epithelial cells. Data for the expression of specific genes are taken from Table S1. Error bars indicate the standard error of the mean.

Inside macrophages, most changes in the *S.* Typhimurium transcriptome were observed at 4 h p.i. and the expression of very few genes changed at later stages of infection (Eriksson *et al*., 2003; Lucchini *et al*., 2005). We therefore used the 4 h time point in macrophages to compare with the intraepithelial transcriptome of *Salmonella* at 2 h and 6 h p.i. (Fig. 2). We classified the genes that were differentially regulated in both cell types into functional categories (Fig. 3). The analysis revealed that a substantial pool of *S.* Typhimurium genes were upregulated or downregulated in both epithelial cells and macrophages. This represents the qualitative intracellular transcriptomic signature of *S.* Typhimurium, which is apparent from the intersecting areas of Fig. 2A and B and is detailed in Tables S6 and S13. This revealed that certain functional categories of genes showed the same expression profiles in both cell types (Figs 2A, B, 3A and B, Tables S6 and S13), suggests that many common environmental cues exist within both cell types, and have similar impacts upon *S.* Typhimurium gene expression. We note that, although the same categories of genes are changing in both cell types compared with LB (Fig. 3A and B), the level of expression is often different in each cell type. For example, the *mgtBC* and *pstACS* magnesium and phosphate transport systems showed upregulation in both cell types, indicative of a common lack of magnesium and phosphate in both SCVs. Interestingly, while the levels of expression of the *mgtBC* genes remained high in macrophages at all three time points, both genes were substantially downregulated inside epithelial cells between the 2 h and 6 h time points (Fig. 4A, Table S1). This could imply that *S.* Typhimurium utilizes alternative ways of acquiring magnesium, or that the requirement for *Salmonella* to actively transport magnesium changes later in infection. Alternatively, the availability of magnesium to *Salmonella* could change at later stages of infection.

**Fig. 7:**
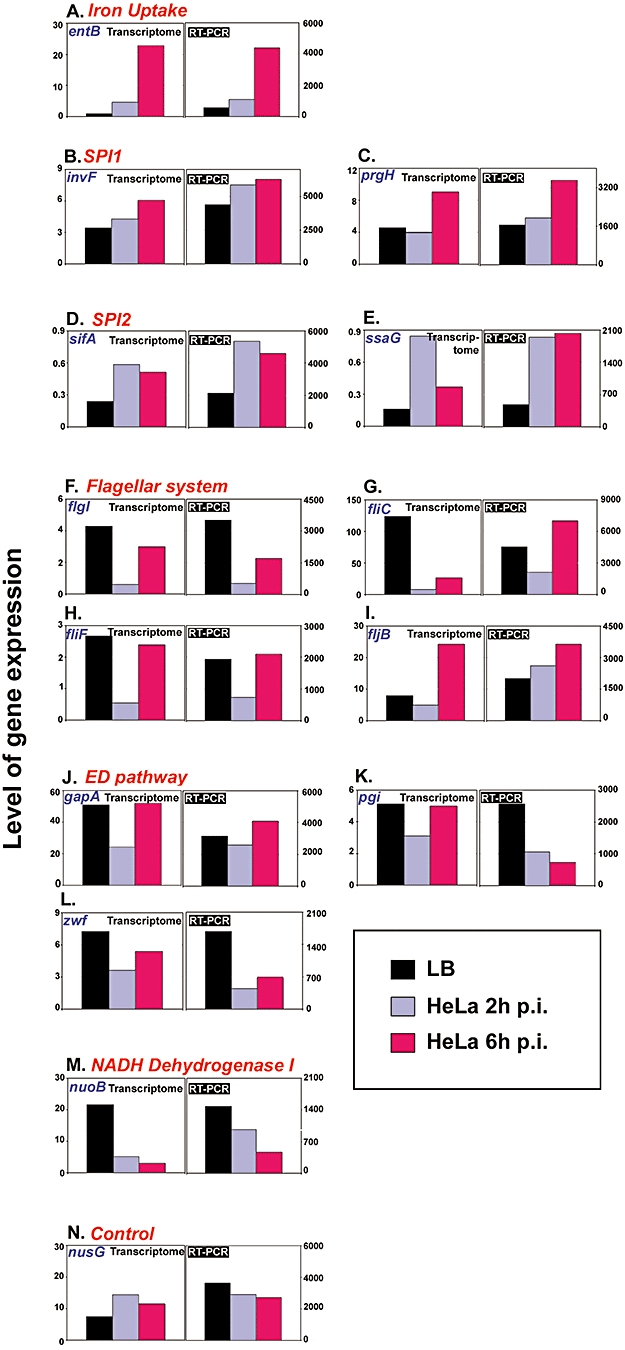
RT-PCR confirmation of transcriptomic data. RNA was extracted from *Salmonella* cells released from infected epithelial cells at 2 and 6 h p.i. and from mid-exponential LB cultures, reverse transcribed to cDNA and used as template for RT-PCR amplification of *entB* (A), *invF* (B), *prgH* (C), *sifA* (D), *ssaG* (E), *flgI* (F), *fliC* (G), *fliF* (H), *fljB* (I), *gapA* (J), *pgi* (K), *zwf* (L), *nuoB* (M) and *nusG* (N) cDNAs using specific primers pairs (Table 5 and *Experimental procedures*). Each panel shows the expression levels observed from the transcriptomic data (graph on the left) and the RT-PCR analyses (graph on the right). Black bars show expression levels determined from LB culture. Purple and magenta bars show expression levels obtained inside epithelial cells at 2 and 6 h p.i. respectively.

Within both cell types, we noted a strong induction of the *ent* (Fig. 7A), *fep*, *fhu*, *iro* (Fig. 4A), *sit* and *suf* genes compared with LB (Table S1). Many of these metal acquisition systems import iron, which forms an essential component of respiratory enzymes which contain iron-sulfur clusters (Beinert *et al*., 1997; Tokumoto *et al*., 2002) and regulatory proteins such as FUR (Tsolis *et al*., 1995). Late infection of epithelial cells (6 h p.i.) showed a 100-fold greater level of expression of these iron uptake systems than the early time point (2 h p.i.), whereas their expression profiles remained similar at all time points inside macrophages. Induction of these bacterial iron-uptake systems within both macrophages and epithelial cells was also observed for *Shigella flexneri*, which has a cytosolic lifestyle inside mammalian cells (Runyen-Janecky and Payne, 2002; Lucchini *et al*., 2005), suggesting that uptake of iron is triggered in both the macrophage cytosol for *Shigella* and within the SCV for *Salmonella*. Additionally, we observed that the *sfbAB* genes, encoding the periplasmic iron-binding lipoprotein SfbA and the nucleotide-binding ATPase SfbB, are up to sixfold more highly expressed in epithelial cells than in macrophages. The *sfbABC* operon has been identified as a pathogenicity islet in *S. enterica* sv. Enteritidis, important for ferric iron acquisition in BALB/c mice (Pattery *et al*., 1999). Overall, our observation of the upregulation of both ferrous and ferric iron uptake systems suggests that *Salmonella* is actively acquiring iron inside epithelial cells.

**Table 5:** Oligonucleotides used in this study.

Name	Sequence	Use in this study
bio_F	CTTGCCCGACGAAGAAGCGTTGAAGCGCCGCCATATTGACCGGATGCGTG gtgtaggctggagctgcttc^a^	Construction of deletion mutant
bio_R	AAAAACAGCAGGATCGTCTGATCCTGCAAGAAGGCAGTTTTTAAGCGCAGcatatgaatatcctccttag^a^	Construction of deletion mutant
uhpT_F	TTTACAATGCCTGCCATTCGCAGGTATAAAAATTAGCTCAGGAGTAATCCgtgtaggctggagctgcttc^a^	Construction of deletion mutant
uhpT_R	GGCGTCCAGCGCGGCGAACGTGCCCGCCCAGCCGGTCAGACCAAAAACGGcatatgaatatcctccttag^a^	Construction of deletion mutant
fliC-3xFlag_F	GGCGAACCAGGTTCCGCAAAACGTCCTCTCTTTACTGCGTgactacaaagaccatgacgg^a^	Construction of FliC-3xFlag protein
fliC-3xFlag_R	CCTTGATTGTGTACCACGTGTCGGTGAATCAATCGCCGGAcatatgaatatcctccttag^a^	Construction of FliC-3xFlag protein
entB RT_F2	gtttatgcgcgacattaagc	RT-PCR confirmatory experiments
entB RT_R2	ccatcatgcgtactgaatcc	RT-PCR confirmatory experiments
flgI RT_F2	ggcggacattcaaaatatgg	RT-PCR confirmatory experiments
flgI RT_R2	acatcagatccatcggcgtc	RT-PCR confirmatory experiments
fliC RT_F2	tgacagcagcaggtgttacc	RT-PCR confirmatory experiments
fliC RT_R2	gttgtgaccttcggctttac	RT-PCR confirmatory experiments
fliF RT_F2	gaatgtgggcgatattgagc	RT-PCR confirmatory experiments
fliF RT_R2	tgttgccagaagggcagttc	RT-PCR confirmatory experiments
fljB RT_F2	ggtaagacaattgaaggcgg	RT-PCR confirmatory experiments
fljB RT_R2	ccagctctggttgtgctttg	RT-PCR confirmatory experiments
gapA RT_F2	tactccgaacgtatccgttg	RT-PCR confirmatory experiments
gapA RT_R2	tcgaacacggaagtgcatac	RT-PCR confirmatory experiments
invF RT_F2	aagatcgtaaacgctgcgag	RT-PCR confirmatory experiments
invF RT_R2	attgggtgatgttctcgtgg	RT-PCR confirmatory experiments
nuoB RT_F2	tatgaccaaatgctggagcc	RT-PCR confirmatory experiments
nuoB RT_R2	tgccaatcgattcttgcagc	RT-PCR confirmatory experiments
nusG RT_F2	tggtccagatggtcatgaac	RT-PCR confirmatory experiments
nusG RT_R2	ggcgagacttctcataatcg	RT-PCR confirmatory experiments
pgi RT_F	gggtaacatggaatccaacg	RT-PCR confirmatory experiments
pgi RT_R	taccctgatcgcgatattcc	RT-PCR confirmatory experiments
prgH RT_F	gagatacgttgtgggctcgt	RT-PCR confirmatory experiments
prgH RT_R	gcgctctcagcttttgactt	RT-PCR confirmatory experiments
sifA RT_F	ccatgcgaatatatccgaaa	RT-PCR confirmatory experiments
sifA RT_R	aaaatggcgtgaaaaacctg	RT-PCR confirmatory experiments
ssaG RT_F2	aggccaggccattaatgaca	RT-PCR confirmatory experiments
ssaG RT_R2	tagcaatgattccactaagc	RT-PCR confirmatory experiments
zwf RT_F2	tcaagacgcctgaactgaac	RT-PCR confirmatory experiments
zwf RT_R2	cttcggtaatggagtccacc	RT-PCR confirmatory experiments

a Uppercase sequences indicate homology with the flanking regions of the target genes or operon.

The SCVs of both macrophage and epithelial cells have been reported to be limiting for purines, pyrimidines and aromatic amino acids (Finlay *et al*., 1991; Leung and Finlay, 1991). Consistent with these data, we observed that expression of the genes involved in *de novo* biosynthesis of purines was induced up to sevenfold in both macrophages and epithelial cells (Table S1).

### Differences in *Salmonella* Typhimurium gene expression within the SCV of epithelial and macrophage cells

A direct comparison between the *S.* Typhimurium transcriptomes in epithelial cells (2 and 6 h p.i.) and macrophages (4 h p.i.) identified 627 and 1032 genes, respectively, that were up- or downregulated by more than twofold (Table 1). This suggests that *S.* Typhimurium gene expression within the macrophage SCV is most similar to the early epithelial SCV (2 h p.i.) (Fig. 2). We also identified clusters of genes that showed epithelial cell-specific or macrophage-specific patterns of expression (Fig. 2); 269 and 166 genes are upregulated or downregulated in epithelial cells compared with LB but not in macrophages, respectively (Fig. 2A and B, Tables S3–S5 and S10–S12). In contrast, 345 and 333 genes were up- or downregulated in macrophages but not in epithelial cells (Fig. 2A and B, Tables S9 and S16).

Subsequent time-dependent changes in the *S.* Typhimurium transcriptome occurred at 6 h p.i. in epithelial cells, with expression of 187 genes changing at 6 h compared with 2 h p.i. (Table 1). Among these differences, genes encoding the superoxide dismutase (*sodBC*), alkyl hydroxyperoxidase (*ahpCF*), catalase (*katE*) and flavohemoglobin (*hmpA*) showed low levels of expression, reflecting the lack of oxidative or nitrosative stress in HeLa cells (Table S1; Fig. S1). The main differences are described and discussed in more detail below.

#### Biotin synthesis

We noted that biotin synthesis genes were highly induced in epithelial cells, showing up to 10-fold higher expression than in macrophages (Fig. 4B). Biotin enzymes function as carboxyl group transfer enzymes, and are necessary for fatty acid biosynthesis (Keseler *et al*., 2005). Induction of *Sh. flexneri* biotin biosynthesis genes were also observed during infection of epithelial cells but not macrophages (Lucchini *et al*., 2005), suggesting differences in the availability or importance of biotin during infection of these mammalian cells. We investigated the role of the *bio* genes by infecting epithelial cells with a *bioABFCD* deletion mutant of *S.* Typhimurium. Although, this gene deletion prevented growth of the mutant on minimal medium that lacked biotin, the strain was not attenuated in its ability to invade or replicate within epithelial cells compared with its parental strain SL1344 (Table 3). This suggests that biotin synthesis is not crucial for the survival and replication of *S.* Typhimurium inside cultured epithelial cells. Differences in the induction of biotin biosynthesis genes by *Salmonella* during infection of epithelial cells and macrophages could reflect fluctuations in the intracellular availability of biotin, or that other signals trigger *bio* gene expression inside epithelial cells.

**Table 3:** Genes involved in biotin utilization of hexose phosphate utilization are not required for intracellular replication of *S*. Typhimurium.

Strains	Genotype	Invasion^a^(% inoculum)	Change in intracellular population from 2 to 6 h p.i. compared with wt^b^
SL1344	wt	18 ± 1.15	1
JH3214	SL1344Δ*bio*	25 ± 1.76	0.93 ± 0.09
JH3216	SL1344Δ*uhpT*	34 ± 1.20	0.88 ± 0.04

a Invasion ability is the ratio of the intracellular bacterial cell number at 2 h p.i. over that of the inoculum (± standard error of the mean).

b Ratio of change in intracellular bacterial numbers at 6 h compared with 2 h p.i. for each mutant over that of wt SL1344 strain.

#### Intra-vacuolar pH

Upregulation of acid-inducible *S.* Typhimurium genes, such as *pagC* (Alpuche Aranda *et al*., 1992), *cysB*, *adiY*, *marAB* inside the macrophage vacuole is consistent with the acidic nature of the SCV (Rathman *et al*., 1996; Eriksson *et al*., 2003). Inside epithelial cells, expression of four of these five acid-inducible genes was either downregulated or did not change compared with LB. Only the *cysB* transcriptional activator showed a fourfold to sevenfold induction in this cell type (Table S1). These data suggest that the macrophage SCV is more acidic than the epithelial SCV.

#### Cell type-specific expression of FUN genes

*S.* Typhimurium SL1344 carries over 900 genes of unknown function (FUN genes (Hinton, 1997)). Expression of about 30% of these genes was altered inside epithelial cells compared with LB. These genes are designated as ‘putative’ or ‘hypothetical’ in our Supplementary Tables. Thirty-two of these genes were only upregulated in epithelial cells at 2 h p.i. and not in macrophages, whereas 96 genes were only induced in macrophages after 4 h infection and not in epithelial cells. One hundred and twenty-eight FUN genes were upregulated in both cell types (Table S1). The FUN genes that show cell type-specific expression may reveal new virulence determinants, and are currently under investigation.

### All three T3SS are upregulated inside epithelial cells

Focusing on the T3SS of *Salmonella* Typhimurium such as SPI1, SPI2 and the flagellar system, we observed major differences in expression profiles between the two host cell types. The role of the three T3SS of *Salmonella* is often perceived as follows: flagella genes are seen as being important for the extracellular life of *Salmonella*, and for reaching the epithelial barrier, while SPI1 mediates the invasion process and SPI2 ensures intracellular survival. It might have been thought that sequential expression of the T3SS would facilitate successful infection but we have now shown that all three T3SS are upregulated simultaneously inside epithelial cells.

#### SPI1 genes are expressed inside epithelial cells

**Fig. 5:**
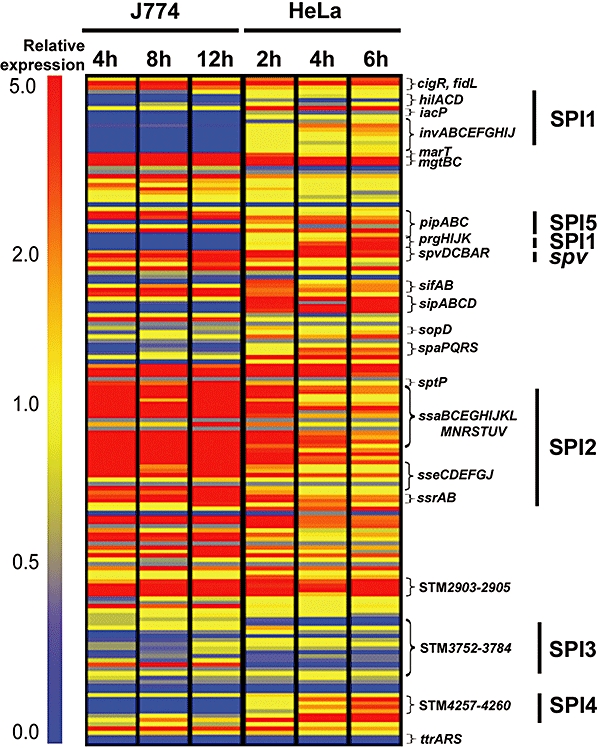
SPI1 and SPI2 genes are upregulated inside epithelial cells. The expression profiles of SPI1 to SPI5 genes, their known effectors (Abrahams and Hensel, 2006; McClelland *et al*., 2001), and the *spvABCDR* virulence plasmid genes inside macrophage and epithelial cells are shown relative to the LB comparator. Each horizontal bar represents the expression level of a single gene. The post-infection sampling time is indicated. Blue shows that the gene is downregulated, yellow that it is expressed at similar levels and red that it is upregulated, compared with LB. Data are taken from Table S1, and are summarized in Table S2.

SPI1 and SPI1-associated genes are necessary for the invasion of mammalian cells, for full virulence in orally infected mice or infected murine ileal loops, and for causing enteritis in bovine and porcine infection models (Penheiter *et al*., 1997; Barthel *et al*., 2003; Morgan *et al*., 2004; Stecher *et al*., 2005; Carnell *et al*., 2007). We have previously shown that SPI1 structural genes are strongly downregulated inside macrophages at 4 h p.i. (Eriksson *et al*., 2003), and under *in vitro* conditions that mimicked the environment of the macrophage SCV (Eriksson-Ygberg *et al*., 2006). Surprisingly, SPI1 genes remained expressed not only once *S.* Typhimurium was inside the epithelial SCV (at 2 h p.i.), but some of these genes were actually upregulated at 4 and 6 h p.i. compared with 2 h p.i. (Figs 5, 7B and C, Table S2). This suggests that the intracellular environment encountered by *Salmonella* at late infection inside epithelial cells contains more SPI1-inducing cues than at earlier stages. As observed in macrophages (Eriksson *et al*., 2003), SPI4 and some SPI5 genes such as *pipC* (Fig. 5), *sopB*, *orfX*, STM1089 and STM1093 (Table S1) were coexpressed with SPI1 genes in epithelial cells. Intracellular expression of SPI1 would be consistent with the additional roles of SPI1 and SPI1-related proteins in interfering with the host response during the later stages of infection (Murray and Lee, 2000; Drecktrah *et al*., 2005). Furthermore, SPI1 genes are known to contribute to the induction of the host pro-inflammatory response involving the recruitment of phagocytes into the gut mucosa (Galyov *et al*., 1997; Jones *et al*., 1998; Gewirtz *et al*., 1999).

#### SPI2 genes are expressed in both macrophages and epithelial cells

The SPI2 island is crucial for the intracellular survival and replication of *S.* Typhimurium in phagocytic and non-phagocytic cells (Hensel, 2000). SPI2 effectors and structural proteins are necessary for the trafficking of the SCV (Salcedo and Holden, 2003; Guignot *et al*., 2004), for the recruitment of the appropriate endosomal markers and for the maintenance of integrity of the SCV membrane (Beuzon *et al*., 2000; Beuzon *et al*., 2002). In macrophages, SPI2 gene expression was strongly induced and remained induced at all time points (Eriksson *et al*., 2003). As expected, SPI2 genes and the SsrA regulon (Rytkonen *et al*., 2007) were also upregulated within epithelial cells. We noticed that the levels of expression of SPI2 and SPI2-related genes were similar to those in macrophages early during epithelial infection (2 h p.i.), but were slightly reduced at subsequent time points (4 and 6 h p.i.) (Figs 5, 7D and E; Table S2). As observed in macrophages (Eriksson *et al*., 2003), some SPI5 genes such as *pipABD* showed similar expression patterns to SPI2 genes inside epithelial cells, consistent with the important role of SPI5 in intracellular survival (Ehrbar *et al*., 2002). We hypothesize that SPI2 expression inside epithelial cells results from the low level of iron or phosphate present in the host cells, because SPI2 genes have been reported to be upregulated under low iron- and low-phosphate conditions (Zaharik *et al*., 2002; Lober *et al*., 2006).

#### *S*. Typhimurium SL1344 produces flagellin inside epithelial cells

The importance of flagella in pathogenesis has long been established for various bacteria (Achtman *et al*., 1986; Akerley *et al*., 1992; Legnani-Fajardo *et al*., 1996; Feldman *et al*., 1998; Nuijten *et al*., 2000) but flagella have become increasingly relevant to the study of intravacuolar pathogens, such as *Legionella* and *Salmonella* (Amer *et al*., 2006; Franchi *et al*., 2006; Miao *et al*., 2006; Miao *et al*., 2007). Although it was initially believed that flagella merely accelerated the invasion process of *Salmonella* (van Asten *et al*., 2004), studies have shown that lack of flagella caused attenuation at several different levels during infection (Schmitt *et al*., 2001). Furthermore, recent studies propose that, in some circumstances, *Salmonella* secretes flagella from the SCV into the cytosol of macrophages, in a SPI1 T3SS-dependent manner (Miao *et al*., 2007; Sun *et al*., 2007). It has been suggested that intracellular flagellin secretion has at least two purposes: first, cytosolic recognition of flagellin through the NLR-mediated signalling pathway would contribute to trigger a pro-inflammatory response (Sun *et al*., 2007); second, production of flagellin inside mammalian cells could lead to the escape of *Salmonella* from dying macrophages (Sano *et al*., 2007). These observations suggest that the flagellin protein plays a direct role in *Salmonella* pathogenesis, which is independent of the role of flagella in motility. However, we note that this phenomenon was only observed when macrophages were infected with *Salmonella* grown in SPI1-inducing conditions. When *Salmonella* were grown in non-SPI1 inducing conditions, we did not observe *de novo* transcription of *fliC* and *fljB* flagellin-encoding genes inside macrophages (Eriksson *et al*., 2003).

**Fig. 6:**
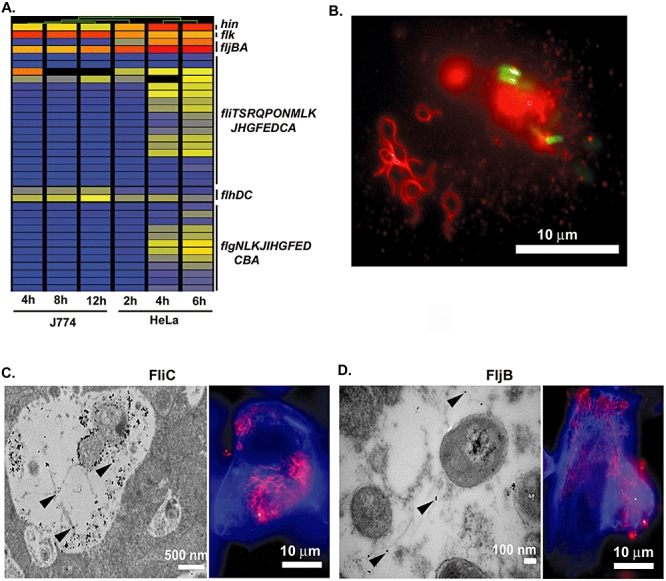
Expression of flagella by *S*. Typhimurium SL1344 inside epithelial cells. Cluster diagram of the expression profile of 36 flagella genes inside macrophage and epithelial cells, relative to the LB comparator (A). The colours are as Fig. 5A. Fluorescence micrograph shows polymerized flagella (red) separated from *ssaG::gfp*+ expressing JH3009 *Salmonella* cells (green) within epithelial cells at 6 h p.i. (B). Fluorescence and Transmission immunogold electron micrographs showing presence of FliC and FljB inside epithelial cells at 6 h p.i. (C, D). Flagella proteins were visualized with Anti-FliC (C) and Anti-FljB (D) antibodies. The arrow heads show immunogold labelling of FliC (C) and FljB (D) on TEM micrographs. The fluorescence micrographs in panels C and D show actin (blue) and large amounts of intracellular flagellin (magenta).

Our protocol involved the infection of epithelial cells with bacteria which had been grown in SPI1-inducting conditions; the transcriptomic analysis revealed time-dependent upregulation of *S.* Typhimurium flagella genes. Over 50 genes are required for a functional flagellar system (Chilcott and Hughes, 2000; Macnab, 2007). In macrophages, most flagellar genes were downregulated and remained downregulated at all time points (see Fig. 6A) (Eriksson *et al*., 2003). A similar pattern of expression was observed inside epithelial cells after 2 h infection when most flagella genes were also downregulated (Fig. 6A). Strikingly, the expression of 33 flagella genes were subsequently upregulated after 4 and 6 h infection of epithelial cells, reaching similar levels of expression to those seen in LB. Immunogold and immuno-fluorescent labelling against the FliC and FljB flagellins showed that *fliC*- and *fljB*-encoded proteins are present at detectable levels in infected epithelial cells at 6 h p.i. (Fig. 6C and D). The fluorescent micrograph on Fig. 6B shows discrete polymerized *Salmonella* flagella within infected cells. This was confirmed by confocal microscopy (data not shown).

#### Verification of *de novo* production of flagellin and SPI2 protein within epithelial cells

In order to support our observation concerning *fliC* and *fljB* expression, we tested transcription of flagellar genes by RT-PCR, and observed increased flagellar gene expression at 6 h p.i. inside epithelial cells (Fig. 7F–I; Table S1).

**Fig. 8:**
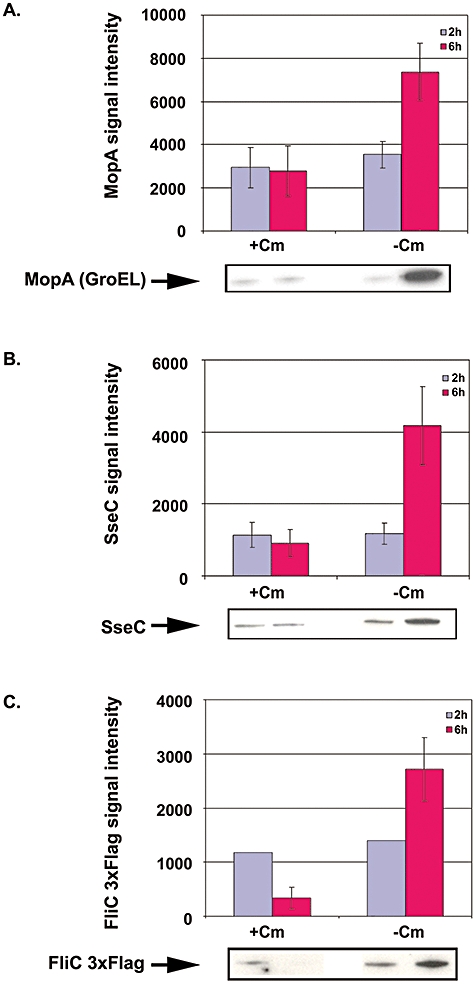
Confirmation of the *de novo* production of MopA, SseC and FliC 3xFlag by *S*. Typhimurium SL1344 inside epithelial cells. The levels of MopA (GroEL) (A), SseC (B) and 3xflag FliC (C) proteins detected at 2 h (purple) and 6 h (magenta) post infection in lysates of infected epithelial cells. *De novo* protein synthesis was assessed by treating intracellular bacterial cells with chloramphenicol and comparing protein levels with that of untreated intracellular bacteria. Experiments were performed in triplicate. The error bars indicate the SEM. For each protein, a representative example of the Western blot is given below the bar chart.

As the detection of flagella proteins inside epithelial cells could have resulted from flagellin transcytosis (Lyons *et al*., 2004), we tested *de novo* synthesis of flagellin by assessing the levels of flagellin protein throughout infection in the presence or absence of chloramphenicol to inhibit protein synthesis (*Experimental procedures*). We first confirmed that bacterial protein synthesis had been inhibited intracellularly by quantifying MopA (GroEL) at 2 h and 6 h p.i. MopA was selected because transcription levels of the *mopA* gene remain fairly constant at all time points inside epithelial cells and in LB broth culture (Table S1). When protein synthesis was active, twice as much MopA was detected at 6 h p.i. compared with the 2 h time point. When bacterial protein synthesis was inhibited, the amount of MopA was similar at both time points (Fig. 8A), confirming that bacterial protein synthesis was effectively inhibited by chloramphenicol inside mammalian cells. Similar results were observed for the SPI2 protein SseC, a *Salmonella* protein that is synthesized inside mammalian cells (Fig. 8B). A greater than threefold increase was observed in SseC between 2 h and 6 h p.i. inside epithelial cells, and this increase was dependent upon protein synthesis. This confirmed that the SPI2 apparatus was synthesized *de novo* inside epithelial cells. We note that the increase of the total amount of SseC and MopA reflects the level of intracellular bacterial replication.

This system was used to show that flagellin was produced *de novo* inside epithelial cells (Fig. 8C). A twofold increase of FliC 3xFlag was detected inside epithelial cells at 6 h p.i. compared with the 2 h time point, and this was dependent upon bacterial protein synthesis. When bacterial protein synthesis was inhibited, around three times less FliC 3xFlag was detected at 6 h than at 2 h p.i. (Fig. 8C), whereas the level of the MopA control remained stable at the two time points (Fig. 8A). The reduced amount of intracellular flagellin at 6 h could result from degradation of FliC in the intraepithelial environment where substantial proteolysis may occur; MopA would be less exposed to the host proteolytic action of the host cell as it remains inside the bacterial cells. The twofold increase of FliC 3xFlag is probably an underestimation of the intracellular production of FliC, as it represents both proteolytic degradation of FliC and *de novo* synthesis. Clearly, this increase reflects bacterial intracellular replication, and shows for the first time that flagellin is produced within epithelial cells at this stage of infection.

The induction of expression of a *fliC::gfp* transcriptional gene fusion has been reported during murine infection (Cummings *et al*., 2006). The authors noted that *fliC::gfp* transcription was restricted to an unknown cell type located in Peyer's patches, and was not observed either in the mesenteric lymph nodes or in the spleen of infected mice. Our study builds on the work of Cummings *et al*. and provides the first description of *de novo* production of flagella by *S.* Typhimurium inside epithelial cells. It is not clear why *Salmonella* should do this, but it is conceivable that the flagellar T3SS could secrete other proteins including virulence effectors, as has been shown for other pathogens such as *Yersinia enterocolitica* (Young *et al*., 1999) and postulated to occur in *Salmonella* (Komoriya *et al*., 1999). Alternatively, production of flagellin could reflect bacterial adaptation to the fate of the host cell (see below).

#### *S*. Typhimurium SL1344 is highly cytotoxic to epithelial cells

**Fig. 9:**
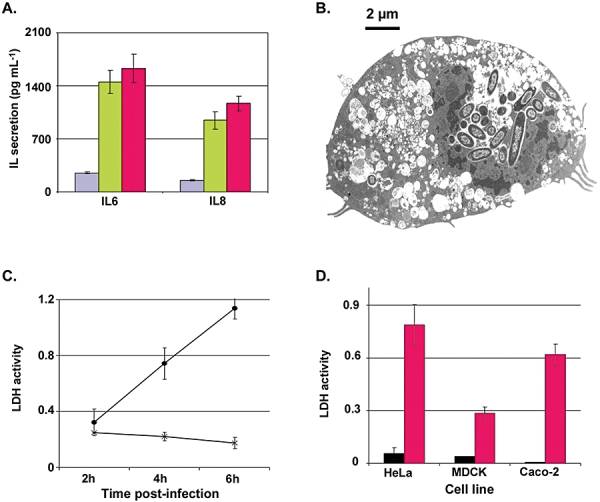
*S*. Typhimurium is cytotoxic to epithelial cells. Levels of IL-6 and IL-8 secreted by HeLa cells infected with SL1344 were measured by flow cytometry (*Experimental procedures*). Levels of secreted IL from epithelial cells at 2 h (purple), 4 h (green) and 6 h (magenta) post infection (A). Error bars in panels A, C and D show SEM. Transmission electron micrograph showing a dying epithelial cell at 6 h post infection which was representative of 70% of the infected epithelial cell sections that were observed (B). Cytotoxic effect of SL1344 strain (closed circle) on epithelial cells after 2, 4 and 6 h infection compared to non-infected cells (cross) (C). All cytotoxicity data were obtained from three independent replicates. Cytotoxic effect of SL1344 strain on HeLa, MDCK and Caco-2 epithelial cell lines. Black bars show LDH levels obtained on uninfected epithelial cells. Magenta bars show the LDH levels obtained on infected epithelial cells after 6 h infection (D).

The intracellular production of three different T3SSs was unexpected and may reflect a difference in the morphology of the epithelial cells between early and late infection stages. The epithelial cells responded promptly to SL1344 infection by inducing nearly twice as much IL-6 and IL-8 secretion at 2 h p.i. compared with uninfected cells. By 6 h p.i., the level of secretion of both pro-inflammatory cytokines was over 10-fold higher than in non-infected cells (Fig. 9A). Inhibition of bacterial protein synthesis did not change the level of release of IL-6 and IL-8 upon infection (data not shown), suggesting that cytokine secretion is induced at an early stage of *Salmonella* infection. We performed microscopic analysis of *Salmonella*-infected epithelial cells, which revealed striking cytotoxic effects. At 6 h p.i., TEM and light microscopic observations showed that 70% of the epithelial cells showed signs of cell death, such as chromatin condensation or vacuolization of the cell cytoplasm (Zychlinsky and Sansonetti, 1997; Fink and Cookson, 2005) (Fig. 9B). This alteration in cell morphology coincided with an increased release of the cytoplasmic lactate dehydrogenase (LDH) into the culture medium at 4 and 6 h p.i. (Fig. 9C). Such cytotoxicity by *S.* Typhimurium SL1344 was not restricted to HeLa epithelial cells, but also observed upon infection of two other epithelial cell lines, MDCK and Caco-2 (Fig. 9D). At similar time points, infected J774 A.1 macrophage cells retained their integrity (data not shown).

The substantial and rapid cytotoxicity caused by *S.* Typhimurium SL1344 in epithelial cells leads us to suggest a two-step hypothesis to explain the temporal change in *Salmonella* gene expression patterns that were observed. During the early adaptation of *Salmonella* to the epithelial SCV a component of the transcriptomic changes could lead to cytotoxicity. This cell death might serve to moderate epithelial cell pro-inflammatory cytokine responses. Second, the time-dependent changes in the intraepithelial transcriptome of *S.* Typhimurium SL1344, such as the fluctuations in expression of *mgtBC*, iron uptake systems and flagella genes (Figs 4 and 6), could reflect the response of *Salmonella* to the increasing levels of epithelial cell death.

### Expression of T3SS is associated with increased carbohydrate metabolism and energy requirement by *Salmonella* inside epithelial cells

The simultaneous expression of the three T3SS inside epithelial cells is a remarkable event which raises several questions that are currently under investigation. We also hypothesize that such a drain on cellular energy levels would be reflected in the expression patterns of the main metabolic pathways. In *E. coli*, the demand for ATP controls over 75% of the glycolytic flux (Koebmann *et al*., 2002). Our transcriptomic data showed that genes involved in ATP synthesis were transcribed at higher levels in epithelial cells than in macrophages (Table S1). These observations suggest that more glucose is catabolized by *Salmonella* inside epithelial cells than in macrophages. Elevated glucose metabolism of intraepithelial *Salmonella* also correlates with the upregulation of *ptsG* and *uhpT* genes (Fig. 4B), encoding a high affinity glucose transporter (Melton *et al*., 1976) and a hexose phosphate transporter, respectively (Table S1), suggesting that both sugars and sugar phosphates might be available to intraepithelial *Salmonella*. A similar observation was made for *Listeria monocytogenes* and *Shigella flexneri* (Chico-Calero *et al*., 2002; Lucchini *et al*., 2005). When *Salmonella* lacked the *uhpT* gene, it showed reduced growth on glucose 6-phosphate as sole carbon source *in vitro* (data not shown); however, no reduction in growth was observed in epithelial cells compared with the parental strain (Table 3), as observed for *Shigella* (Runyen-Janecky and Payne, 2002), suggesting either that glucose 6-phosphate is not the only available intracellular carbon source or that it is transported by other sugar transporters that are induced intracellularly but not *in vitro* (Table S1).

**Fig. 10:**
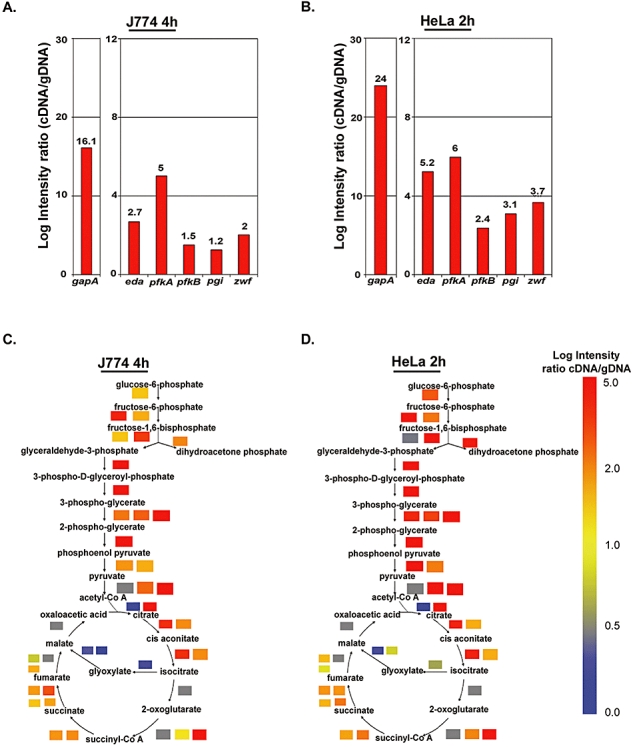
The *S.* Typhimurium TCA cycle, Entner–Doudoroff and Glycolytic pathways are more active inside epithelial cells than macrophage cells. Panels A and B show expression levels of *S*. Typhimurium genes involved in the Entner–Doudoroff pathway inside macrophages at 4 h p.i. (A) and inside epithelial cells at 2 h p.i. (B). These bar charts show the approximate level of each mRNA from the un-normalized transcriptomic data set. C and D show colour-coded expression levels for genes involved in each step of the *S.* Typhimurium glycolysis and TCA pathways. This figure does not involve normalization to a comparator but uses transcriptomic data to show the approximate level of each mRNA from the non-normalized Log intensity ratios between cDNA and genomic reference DNA. Each coloured block refers to a specific gene that encodes a relevant enzyme involved in particular metabolic pathways. Red indicates that the gene is highly expressed, yellow that the gene is expressed at intermediate levels, blue that the gene is poorly expressed and grey that no data were available for that gene. Panel C shows the gene expression data from *Salmonella* isolated from macrophages (4 h p.i.) and (D) data obtained from *Salmonella* isolated from epithelial cells (2 h p.i.). Key differences can be seen by comparing particular coloured blocks across C and D.

Many *Salmonella* genes encoding enzymes of the Entner–Doudoroff (ED) pathway were expressed in both epithelial cells (Fig. 7J–L) and macrophages (Table S1), with genes such as *zwf*, *pgi* and *gapA* showing up to twofold higher expression in epithelial cells than in macrophages (Fig. 10A and B). The ED pathway facilitates growth on sugar acids such as galacturonate that are found in the gastrointestinal tract (Salyers and Leedle, 1983; Allen, 1984). Together these data suggest that *S.* Typhimurium may use host-sugar acids as intracellular carbon sources, in both macrophages and epithelial cells, in addition to host-derived glucose in epithelial cells.

TCA cycle and glycolysis genes were expressed in *Salmonella* isolated from both infected macrophages and epithelial cells (Fig. 10C and D; Table S1), implying that both pathways are still active inside these host cells. Our results are consistent with a recent study which detected *Salmonella* enzymes involved in the TCA cycle inside infected spleenic macrophages (Becker *et al*., 2006). We observed that the central metabolic pathway of glycolysis, a major route for glucose catabolism, was expressed at higher levels in infected epithelial cells than in macrophages (Fig. 10C and D, Table S1).

The elevated activity of the *Salmonella* glycolytic and TCA pathways in epithelial cells fits with the higher expression levels of several *Salmonella* genes involved in the aerobic electron transport chain and ATP generation, such as the *nuo* (Fig. 7M), *cyd*, *hem* and *atp* genes (Table S1). The demand for energy production could therefore be expected to be reflected in the metabolic fluxes of intraepithelial *Salmonella*, involving the production of ATP through glycolysis, TCA cycle and ED pathways. In summary, our data suggest that glucose, phosphate sugars and sugar acids may be important carbon and energy sources inside epithelial cells. Energy from the generated ATP could be utilized for iron, phosphate and other transport systems (Kadner, 1996), and for biosynthetic activities. As the *Salmonella* SL1344 strain replicates only twice between 2 and 6 h p.i. inside epithelial cells, the apparently larger demand for ATP synthesis in epithelial cells than in macrophages could also be linked to the higher levels of expression of T3SS and flagellar machineries within the epithelial cells.

## Concluding remarks

By comparing global gene expression of strain SL1344 inside epithelial cells and macrophages (Eriksson *et al*., 2003), we identified the intracellular transcriptomic signature of *S.* Typhimurium. We have also gained insights into the strategies used by this pathogen to adapt to the two main mammalian cell types in which *S.* Typhimurium can replicate. The key *Salmonella* gene expression profiles inside epithelial cells were validated with RT-PCR on additional biological replicates.

**Fig. 11:**
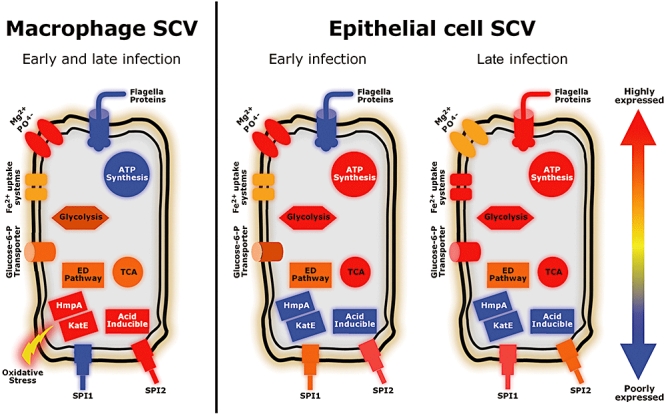
Model proposed for the responses of *Salmonella* Typhimurium to the intracellular environment of macrophages and epithelial cells. The response of *S.* Typhimurium to the macrophage SCV is relatively stable by 4 h p.i. and hardly varies at later time points. Conversely, the epithelial SCV changes through time, probably because *Salmonella* responds to alterations in the epithelial cells from the early to the late stages of infection. Each symbol represents groups of functionally related proteins and is coloured according to expression levels of the appropriate genes under each condition.

Our transcriptomic data are consistent with previous studies that showed that the SCV of both macrophages and epithelial cells contain limiting levels of magnesium, phosphate and iron, and are summarized in Fig. 11. We were interested to see that the genes required for bovine enteritis are highly expressed in HeLa cells (Table S17; Morgan *et al*., 2004). Recently, 95 *S.* Typhimurium genes necessary for porcine intestinal infection were identified (Carnell *et al*., 2007) and these closely parallel the genes implicated in the calf enteritis infection model. Perhaps HeLa cells will be a useful model for the intracellular aspect of epithelial cell-mediated infection, despite the fact that HeLa cells are not differentiated.

Clear differences in virulence gene expression patterns were observed between the macrophages and epithelial cells; for example, oxidative and nitrosative stresses caused higher expression of many detoxification genes inside macrophages than within epithelial cells. This fits with the dogma that epithelial cells are not designed to kill pathogens, and are permissive for bacterial replication.

Genes belonging to the SPI2 and the SsrA regulon were upregulated in both cell types, but their expression levels were higher in macrophages than in epithelial cells (Fig. 11). This expression of SPI2 genes is consistent with the requirement of SPI2 effectors for the biogenesis and evolution of both macrophage and epithelial SCVs, interfering with basic intracellular transport processes such as the host-actin cytoskeleton and microtubule network, as well as the innate and adaptive immune system (Abrahams and Hensel, 2006). The *spvABCDR* genes were also upregulated in both cell types, reflecting the importance of these plasmid-encoded genes for intracellular survival and replication of *S.* Typhimurium (Rhen *et al*., 1993; Gulig *et al*., 1998; Eriksson-Ygberg *et al*., 2006).

Strikingly, SPI1 genes were expressed inside epithelial cells at all time points post invasion, whereas they were strongly downregulated in macrophages (Fig. 11). SPI1 gene products are required for the invasion of mammalian cells but also contribute to the induction of pro-inflammatory responses (Murray and Lee, 2000) and the SPI1 effector protein SopB can stimulate nitric oxide production by macrophages (Drecktrah *et al*., 2005).The SPI1 T3SS machinery can also translocate certain SPI2-dependent effectors, such as SspH1, SlrP or SseK1 (Abrahams and Hensel, 2006). Such cross-talk between SPI1 and SPI2 could occur intracellularly and is consistent with the reported ability of the SPI1 regulator HilA to impact upon transcription of the SPI2 *saaH* gene (Thijs *et al*., 2007).

The second unexpected expression profile involved the flagella biosynthetic genes; these were strongly downregulated inside macrophages at all time points and inside epithelial cells at 2 h p.i., but were induced in a time-dependent manner inside epithelial cells at later stages of infection (Fig. 11). Monomeric flagellin is the ligand of the TLR5 receptor and triggers secretion of the neutrophil chemoattractant IL-8 by epithelial cells (Gewirtz *et al*., 2001b). *S.* Typhimurium flagellin is also transcytosed in a SPI2-dependent manner to the basolateral side of epithelial cells, in a separate compartment to the SCV (Lyons *et al*., 2004). Additionally, flagellin present in the cytosol of infected macrophages can be recognized by the Birc- 1e/Naip5 and Ipaf cytosolic NOD receptor molecules (Inohara *et al*., 2005; Franchi *et al*., 2006; Miao *et al*., 2007), triggering the pro-inflammatory signalling cascade as well as pyroptosis. Recently, monomeric FliC has been shown to be produced and secreted by extracellular *Salmonella* Typhi and Typhimurium upon contact with epithelial cell-derived lysophospholipids (Subramanian and Qadri, 2006). Our results show for the first time that *S.* Typhimurium can produce flagellin *de novo* inside epithelial cells, and the consequent biological implications are currently under investigation. Additionally, it is possible that expression of flagellar genes impacts upon intracellular expression of SPI1 genes, as observed previously *in vitro* (Lucas *et al*., 2000).

The intracellular production of flagellin coincided with a strong cytotoxic effect of *S.* Typhimurium strain SL1344 upon epithelial cells at the late stage of infection. It is possible that the pathogen senses the imminent death of the host epithelial cells and/or the pro-inflammatory signalling mediators, resulting in IL-6 and IL-8 secretion. Upregulation of the genes encoding flagella and the SPI1 T3SS could facilitate the invasion of neighbouring uninfected cells, and might build on the recent report that intracellular flagellin production mediated the escape of *Salmonella* from macrophages undergoing oncosis (Sano *et al*., 2007). It is possible that the SPI1 T3SS could secrete flagellin inside epithelial cells as has been reported in macrophages (Sun *et al*., 2007). This secretion process would be energy demanding and would be consistent with the elevation of *S.* Typhimurium carbon metabolism in epithelial cells compared with macrophages (Fig. 11).

In summary, we have identified likely similarities and differences between the SCVs of epithelial and macrophage cells. Our discovery that all three of the *Salmonella* T3SSs were transcribed simultaneously inside epithelial cells lead us to hypothesize that *Salmonella* is undergoing radical adaptation to epithelial cells that are close to death.

## Experimental procedures

### Bacterial strains and growth conditions

The *S.* Typhimurium SL1344 used in this study, and in our previous transcriptomic analyses (see http://www.ifr.ac.uk/Safety/MolMicro/pubs.html for references), was kindly provided by Catherine Lee (Hoiseth and Stocker, 1981; Shea *et al*., 1999). All strains are listed in Table 4. Strains were grown in 25 ml of LB broth (Sambrook and Russell, 2001) at 37°C, unless stated otherwise. Cultures were shaken in 250 ml flasks at 250 r.p.m. For invasion assays, a 1:10 dilution of a 5 ml overnight bacterial culture was grown in 5 ml of LBS (LB containing a total of 0.3 M NaCl) in 30 ml universals until an optical density at 600 nm (A600) of 1.2. Antibiotics were added as required at the following final concentrations (ampicillin, 100 μg ml^−1^; kanamycin, 50 μg ml^−1^; chloramphenicol, 10 μg ml^−1^).

**Table 4:** Bacterial strains used in this study.

Name	Description	Source orReference
SL1344	4/74 *hisG rpsL*	Hoiseth and Stocker (1981)
JH3009	SL1344 Φ(*ssaG′-gfp*^+^), Cm^r^^a^	Hautefort *et al*. (2003)
JH3214	SL1344 Δ*bioABFCD* (Cm^r^)	This study
JH3216	SL1344 Δ*uhpT*, Cm^r^	This study
JH3053	SL1344 (*fliC-3xflag*), Km^r^^b^	This study
JH3227	SL1344 (pPIR), Amp^r^	This study

a The *gfp* gene fusions are inserted in the *putPA* locus at positions 1 210 040–1211 657 on the LT2 genome (Hautefort *et al*., 2003). For these strains, ‘Φ’ indicates transcriptional gene fusion.

b Carries a protein fusion of the 3xFlag tag to the C-terminal part of FliC.

### Construction of deletion mutants and epitope-tagged chromosomal genes

The entire *bioABFCD* operon and *uhpT* structural genes were replaced by PCR-generated antibiotic resistance cassettes on the *S.* Typhimurium chromosome. Briefly, either a chloramphenicol or kanamycin resistance cassette was amplified by PCR from pKD4 and pKD3 plasmid templates using primers listed in Table 5. Each of these primers includes at its 5′ ends a 40–50 base-long extension showing homology with the flanking regions the target gene. The PCR products were used to replace the coding sequence of the target genes on *S.* Typhimurium chromosome using the Lambda Red recombination system (Datsenko and Wanner, 2000); recombinant strains JH3214 and JH3216 were selected for antibiotic resistance and verified by analytical PCR. Strain JH5053 (Table 3), carrying *fliC* gene tagged with the 3xFlag epitope sequence, was constructed as described previously (Uzzau *et al*., 2001). Recombinant transfer of the 3xFlag sequence into the *fliC* gene was achieved according to the Lambda Red recombination method from Datsenko and Wanner (2000). All deletions or recombinant *fliC* gene were subsequently transduced with P22-phage into a clean *S.* Typhimurium SL1344 background to avoid possible non-intended recombination events (Gemski and Stocker, 1967). Strain JH5053 remained motile, showing that the 3xFlag-tagged flagellin proteins were functional.

### Epithelial cell infection by *Salmonella* strains

Human HeLa and Caco-2 epithelial cells (ECACC, No. 93021013, 86010202, respectively) and MDCK canine epithelial cells (ECACC, No. 85011435) were grown in medium containing DMEM (Cat. D-5796, Sigma) supplemented with 10% Fetal Bovine Serum (FBS; Cat. F-7524, Sigma), 2 mM L-Glutamine (Cat. G-7513, Sigma) and 10 mM HEPES buffer (Cat. H-0887, Sigma). This was subsequently called complete medium.

For the transcriptome analysis of *S.* Typhimurium inside epithelial cells, a total of 10^8^ epithelial cells were seeded in 75 cm^2^ tissue culture flasks (12 flasks in total). For microscopic observations, between 5 × 10^5^ and 10^6^ epithelial cells were seeded in 6-well plates. All epithelial cells were incubated statically at 37°C, 10% CO_2_. *S.* Typhimurium bacteria were grown overnight in LB broth at 37°C, 250 r.p.m. The strains were then subcultured in LBS as described above. The late logarithmic cultures were diluted in complete medium without serum and used to infect the epithelial cell monolayer using a multiplicity of infection of 100 bacteria per epithelial cell. Contact with the epithelial cell monolayer was maximized by 5 min centrifugation at 1500 r.p.m. at room temperature. Infected epithelial cells were immediately incubated for 30 min at 37°C, 10% CO_2_. The time zero of an experiment was set at the beginning of this incubation. Remaining extracellular bacteria were then killed by replacement of the medium with complete medium containing 30 μg ml^−1^ gentamicin. After a 30 min incubation at 37°C, 10% CO_2,_ the gentamicin concentration was reduced to 5 μg ml^−1^ of gentamicin until the end of the assay (i.e. for a further 1, 3 or 5 h).

To assess intracellular bacterial replication in epithelial cells, the SL1344 strain carrying the pPir plasmid (JH3227) was used (Posfai *et al*., 1997; Clements *et al*., 2002). Intracellular replication was assessed as described previously (Eriksson *et al*., 2000). During growth above 30°C, each round of replication results in a proportion of bacterial cells that do not inherit the plasmid, reflecting the level of bacterial replication.

*De novo* bacterial protein synthesis was assessed during epithelial cell infection for strains SL1344wt and JH3053 (Table 3). To inhibit bacterial protein synthesis, chloramphenicol was added to 10 μg ml^−1^ final concentration at the stage where extracellular bacteria are killed in gentamicin-containing medium, 30 min after the start of the assay, until the end of the assay.

### RNA extraction

At each time point, infected epithelial cells were lysed on ice for 30 min in 0.1% SDS, 1% acidic phenol and 19% ethanol in water as described previously (Eriksson *et al*., 2003; Hinton *et al*., 2004). *S.* Typhimurium cells were pelleted after centrifugation of the pooled epithelial cell lysates and RNA was prepared using the Promega SV total RNA purification kit. Bacterial RNA was further purified by an extraction in a 50% acidic phenol-50% chloroform mix. Approximately, 5 × 10^8^ bacteria were isolated from each time point, yielding between 0.5 and 10 μg of total RNA. The transcriptome of SL1344 grown in tissue culture media was considered to be an inappropriate comparator because many infection-relevant and environmentally responsive genes are upregulated in both DMEM and RPMI media, including *mgtC*, *ent*, *fep*, *fhu*, *iro*, *bio*, *pur*, some SPI2 genes and many genes of unknown function (data not shown). Of these genes, the iron-regulated *iroN* genes, the *mgt* magnesium transporter and SPI2 genes were already known to be upregulated inside epithelial and macrophage cells (Garcia-del Portillo *et al*., 1992; Smith *et al*., 1998). Furthermore, a study of *Legionella pneumophila* demonstrated that intracellular nutrition of *Legionella* was independent of the monocytic cell growth conditions and truly reflected the intracellular environment (Wieland *et al*., 2005). We assumed that the best comparator available would be a completely unrelated environment, such as LB broth. Control RNA from *in vitro* grown bacteria was obtained by growing *Salmonella* in LB as described previously (Lucchini *et al*., 2005). Size chromatography of RNA was carried out with an Agilent 2100 Bioanalyser.

### Microarray procedures

Microarray techniques were performed essentially as described before (Lucchini *et al*., 2005; Nagy *et al*., 2006). All our transcriptome analyses were defined using microarrays covering 92% of the genes common between *S.* Typhimurium LT2 and SL1344 strains (Kelly *et al*., 2004).

### Probe preparation and scanning

Because the amount of bacterial RNA extracted from infected epithelial cells were small, RNA were first reverse transcribed into cDNA and subsequently labelled by random priming with Klenow Fragment according to the ‘Labelling protocol for reduced amounts of RNA’ described at http://www.ifr.ac.uk/safety/microarrays/protocols.html

Fluorescently labelled genomic DNA was used as a reference channel for each experiment. Slides were scanned on an Axon 4000A scanner (Axon Instruments) using GenePix version 1.4 software (Axon Instruments). Each hybridization was performed twice. Two biological replicates were performed for the 2 and 6 h time points, and one for the 4 h time point.

### Transcriptomic data analysis

Microarray data analysis was performed as described (Eriksson-Ygberg *et al*., 2006). Spots showing a reference signal lower than background plus two standard deviations or obvious blemishes were excluded from subsequent analyses. Local background was subtracted from spot signals, and fluorescence ratios were calculated using the Genepix version 1.4 software (Agilent). In a few cases, when comparing results from different hybridizations, we observed slight deviations which were dependent on gene expression levels. These were corrected using the Loess function in R (Limma package). To compensate for unequal dye incorporation or any effect of the amount of template, data centring was performed by bringing the median natural logarithm of the ratios for each group of spots printed by the same pin to zero. The complete data set is available as supplementary material. Data that passed the quality controls were analysed using Genespring version GX7.3 software (Agilent). Significance of the centred data at *P* = 0.05 was determined using a parametric-based statistical test adjusting the individual *P*-value with the Benjamini and Hochberg false discovery rate multiple test correction (Benjamini and Hochberg, 1995). We report reproducible changes in gene expression of more than twofold during infection. The LB and HeLa complete data sets have been submitted to the ArrayExpress repository with the accession number E-MEXP-1368.

### Confirmation of transcriptomic data

RT-PCR analyses were performed on transcripts using gene-specific primer pairs (Table 4). Primers were designed *in silico* (http://frodo.wi.mit.edu/cgi-bin/primer3/primer3_www.cgi) to minimize primer–primer complementarity and to yield predicted amplicons in the 100–300 bp range, corresponding to the terminal part of each gene coding sequence. Total *Salmonella* RNA was isolated from bacteria released from infected epithelial cells using the RiboPure-Bacteria RNA purification kit (Ambion), and converted to cDNA by using Stratascript (Stratagene) as described by the manufacturer. The cDNA was used as a template for PCR (PCR Master Mix, Promega). An appropriate number of cycles was chosen for each gene to ensure that the RT-PCR amplification was in the linear range (25–35). RT-PCR products were analysed by agarose gel electrophoresis. The product generated on the *nusG* (also known as *rfaH*) cDNA was used as a reference that shows a low level of variation in expression level in our experiments. PCR product intensities on agarose gels were measured using the AlphaDigidoc AD-1200 densitometry software (Alpha Innotech).

### Fixation of infected mammalian cells for fluorescence microscopy

For fluorescence microscopic observations of infected mammalian cells, samples were immediately fixed for 15 min, at room temperature in 3% paraformaldehyde (w/v) (Sigma) freshly prepared in Phosphate Buffer Saline (PBS) pH 7.2. Fixed samples were subsequently washed in PBS and quenched in PBS containing 10 mM NH_4_Cl overnight at 4°C.

### Fluorescence Immunohistochemistry

The rabbit anti-*Salmonella* FliC and FljB antibodies (Cat. 228 241 and 224 741 respectively, Biosciences Pharmingen) were used at dilution 1:400. Donkey Texas Red-conjugated Anti-Rabbit secondary IgG antibody (Cat. 715 075 150, Jackson Immunoresearch) was used at a final dilution of 1:100. All antibodies were diluted in PBS containing 10% horse serum and 0.1% Saponin. Briefly, samples were washed twice in PBS containing 0.1% Saponin (PBS-0.1% Saponin) and incubated for 1 h at room temperature in the primary antibody solution. The samples were subsequently washed once in PBS-0.1% Saponin and incubated for 1 h at room temperature in the secondary Antibody solution. The coverslips were then washed twice in PBS-0.1% Saponin, once in PBS and finally in H2O. Samples were mounted in Aqua Poly Mount (Cat. 18 606, Polysciences). Samples were observed on a Zeiss LSM510 META laser-scanning confocal microscope or an upright BX51 Olympus Optical Company fluorescence microscope.

### Preparation of samples for Light and Transmission electron microscopy

For immunogold antiflagellin antibody labelling and light microscope observations, *Salmonella*-infected epithelial cells were fixed for 1 h in freshly prepared 4% paraformaldehyde (pH 7). The cells were then harvested and mixed with a drop of 2% low-melting-point agarose (Cat. A-4018, Sigma) near its setting point. The sample was cooled at 5°C for 10 min, chopped into small pieces and then dehydrated in an ethanol series from 10% to 90% with stepwise increases of 10%. After three changes in 100% ethanol, the sample was transferred to 50% LR White resin (London Resin) in ethanol, infiltrated with 100% LR White resin and polymerized at 60°C.

Light microscopic observations were performed on over 100 1 μm thick sections, mounted on glass slides and stained with 1% toluidine blue in 1% borax (pH 11) using a BX60 microscope (Olympus) and Acquis software (Syncroscopy).

For observations of epithelial cell structure, *Salmonella*-infected epithelial cells were briefly fixed in 3% glutaraldehyde (Agar Scientific) in 0.1 M cacodylate buffer (pH 7.2). The cells were subsequently scraped from the plates, pelleted and re-suspended in fresh fixative for 2 h, then washed and stored overnight in buffer alone. Cells were then pelleted and mixed with a drop of 2% low-melting-point agarose near its setting point. Samples were cooled at 5°C for 10 min, chopped into small pieces, then post-fixed in 2% aqueous osmium tetroxide (Agar Scientific) for 2 h and dehydrated in an ethanol series from 10% to 90% with stepwise increases of 10%. After three changes in 100% ethanol, the pieces were transferred to acetone, then infiltrated and embedded in Spurr epoxy resin (Agar Scientific) which was polymerized overnight at 75°C (Spurr, 1969). Sections approximately 75 nm thick were cut with a diamond knife, collected on copper grids, and stained sequentially with uranyl acetate (saturated in 50% ethanol) and Reynold's lead citrate (Reynolds, 1963). Sections were examined and photographed in a JEOL 1200 EX/B transmission electron microscope (TEM) at 80 kV.

### Immunogold labelling

The anti-*Salmonella* FliC and FljB antibodies were used as for fluorescence immunohistochemistry (see above), and were diluted at 1:400. The secondary antibody, goat anti-rabbit conjugated to ultra-small gold (Cat. 800.011, Aurion) was diluted 1:75. Briefly, the free aldehydes in both LR White resin and Spurr-resin sections (previously etched for 10 s in sodium ethoxide, then rinsed in 100% ethanol) and LRW embedded sections, were blocked in 50 mM glycine solution in PBS for 15 min. Sections were subsequently blocked in goat serum blocking solution (Cat. 905.002, Aurion) for 30 min, washed three times for 5 min in acetylated-BSA (BSA-c) buffer (PBS containing 0.1% BSA-c, pH 7.4) and incubated overnight in primary antibody solutions in BSA-c buffer (Cat. 900.099, Aurion). Control sections were incubated in buffer alone. Sections were washed six times for 5 min in BSA-c buffer, and incubated in secondary goat anti-rabbit antibody for 2 h. Sections were washed six times for 5 min in BSA-c buffer, and three times for 5 min in PBS only. Sections were then post-fixed in 2% glutaraldehyde in PBS for 5 min, washed in PBS for 5 min and five times for 2 min in distilled water. Gold labelling was subsequently silver enhanced with Silver R-Gent SE-EM (Cat. 500.033, Aurion), for 25 min. Samples were then washed five times for 2 min in distilled water, stained in uranyl acetate, washed and air dried, before analysis by TEM as above.

### Pro-Inflammatory IL-6 and IL-8 cytokine detection

The quantification of interleukin IL-6 and IL-8 in the supernatants of cultured epithelial cells was determined by using the cytometric bead array for Human IL-6 and IL-8 flex sets (Cat. 558 276 and 558 277 respectively, BD Bioscience, San Jose, CA, USA). The operations were performed according to the manufacturer's instructions. The intensity of the fluorescence signal was acquired on a FACScalibur fluorescence activated cell sorter flow cytometer (BD Becton Dickinson), and analysed using CellQuest software.

### Protein methods

To visualize bacterial proteins by Western blotting, infected epithelial cells were lysed at each time point in 2 ml per well of 0.1% SDS in PBS pH 7.3. Samples were kept at −80°C until further processing. Ten microlitres of each sample was first heated at 100°C for 5 min run on a 12% SDS-PAGE (Cat. NP0342, Invitrogen). Transfer to PVDF membranes was performed as described by the manufacturer (Invitrogen). The membranes were then blocked for 45 min in 10% Marvel Milk prepared in 0.05% PBS-Tween 20. Immunodetection of proteins was performed using a chemiluminescence-based Super signal West Pico Rabbit detection kit (Cat. 34 083, Pierce). When monoclonal primary antibodies were used the goat anti-mouse IgG secondary antibody was used as described by the manufacturer (Cat. 31 430, Pierce). Signal intensity obtained for all proteins detected was compared with that obtained for MopA (GroEL). To increase sensitivity in our Western blot detection method, the C-terminal end of FliC was tagged with 3xFlag. The membrane was subsequently incubated for 1 h at room temperature in a 1:2000 dilution of an Anti-flag mouse monoclonal antibody (Cat. F-3165, Sigma), or a 1:15 000 dilution of an Anti-SseC polyclonal antibody (Kindly provided by Michael Hensel) or a 1:10 000 dilution of an Anti-GroEL polyclonal antibody (Cat. G-6532, Sigma), in 0.05% PBS-Tween 20 containing 5% Marvel milk. Fifteen minutes washes were repeated three times in 0.05% PBS-Tween 20 at room temperature, completed with two additional washes in PBS only after the secondary antibody incubation step. Detection was performed according to the manufacturer's instructions. Protein band signal intensities were measured using the AlphaDigidoc AD-1200 densitometry software (Alpha Innotech).

### Cytotoxicity assay

Epithelial cells were infected as mentioned above except that DMEM without Phenol Red was used to prevent interference with this assay. Furthermore, the FBS content of the complete tissue culture medium was reduced to 5% because of the associated Lactate De-Hydrogenase activity it contains in order to reduce the background level. Lactate De-Hydrogenase enzyme activity released from the mammalian cells was measured in the tissue culture medium using the Cytotox 96 non-radioactive cytotoxicity assay kit (Cat. G1780, Promega) and was considered to reflect cytotoxicity of *S.* Typhimurium.

## Supplementary material

The following supplementary material is available for this article online:

Fig. S1 *Salmonella* is not exposed to oxidative or nitrosative stressors within epithelial cells. Transcriptomic data are shown for the S. Typhimurium *hmpA* and *katE* genes inside both macrophage and epithelial cells. Data for the expression of these genes are taken from Table S1. Error bars indicate the standard error of the mean.

Table S1 The *in vivo* gene expression profile of S. Typhimurium SL1344 strain (4747 gene data set in MS Excel format).

Table S2 Data taken from Table S1, including *Salmonella* Typhimurium known virulence genes, as depicted on Fig. 5.

Table S3 Data taken from Table S1 showing the genes specifically upregulated in HeLa cells at 2 h p.i., as depicted on Fig. 1.

Table S4 Data taken from Table S1 showing the genes specifically upregulated in HeLa cells at 2 and 6 h p.i., as depicted on Fig. 1.

Table S5 Data taken from Table S1 showing the genes specifically upregulated in HeLa cells at 6 h p.i., as depicted on Fig. 1.

Table S6 Data taken from Table S1 showing the genes specifically upregulated in both macrophages and HeLa cells, as depicted on Fig. 1.

Table S7 Data taken from Table S1 showing the genes specifically upregulated in macrophages and only HeLa cells at 2 h p.i., as depicted on Fig. 1.

Table S8 Data taken from Table S1 showing the genes specifically upregulated in macrophages and only HeLa cells at 6 h p.i., as depicted on Fig. 1.

Table S9 Data taken from Table S1 showing the genes specifically upregulated in macrophages at 4 h p.i., as depicted on Fig. 1.

Table S10 Data taken from Table S1 showing the genes specifically downregulated in HeLa cells at 2 h p.i., as depicted on Fig. 1.

Table S11 Data taken from Table S1 showing the genes specifically downregulated in HeLa cells at 2 and 6 h p.i., as depicted on Fig. 1.

Table S12 Data taken from Table S1 showing the genes specifically downregulated in HeLa cells at 6 h p.i., as depicted on Fig. 1.

Table S13 Data taken from Table S1 showing the genes specifically downregulated in both macrophages and HeLa cells, as depicted on Fig. 1.

Table S14 Data taken from Table S1 showing the genes specifically downregulated in macrophages and only HeLa cells at 2 h p.i., as depicted on Fig. 1.

Table S15 Data taken from Table S1 showing the genes specifically downregulated in macrophages and only HeLa cells at 6 h p.i., as depicted on Fig. 1.

Table S16 Data taken from Table S1 showing the genes specifically downregulated in macrophages at 4 h p.i., as depicted on Fig. 1.

Table S17 Genes required for *Salmonella*-associated enteritis in the bovine infection model and for survival and replication in cultivated epithelial cells are expressed within HeLa cells.

This material is available as part of the online article from: http://www.blackwell-synergy.com/doi/abs/10.1111/j.1462-5822.2007.01099.x
