# Supplementary figures and images for: During infection of epithelial cells Salmonella enterica serovar Typhimurium undergoes a time-dependent transcriptional adaptation that results in simultaneous expression of three type 3 secretion systems
Source: Cell Microbiol. 2008 Apr 1;10(4):958–84. doi: 10.1111/j.1462-5822.2007.01099.x (PMC2343689; doi:10.1111/j.1462-5822.2007.01099.x)

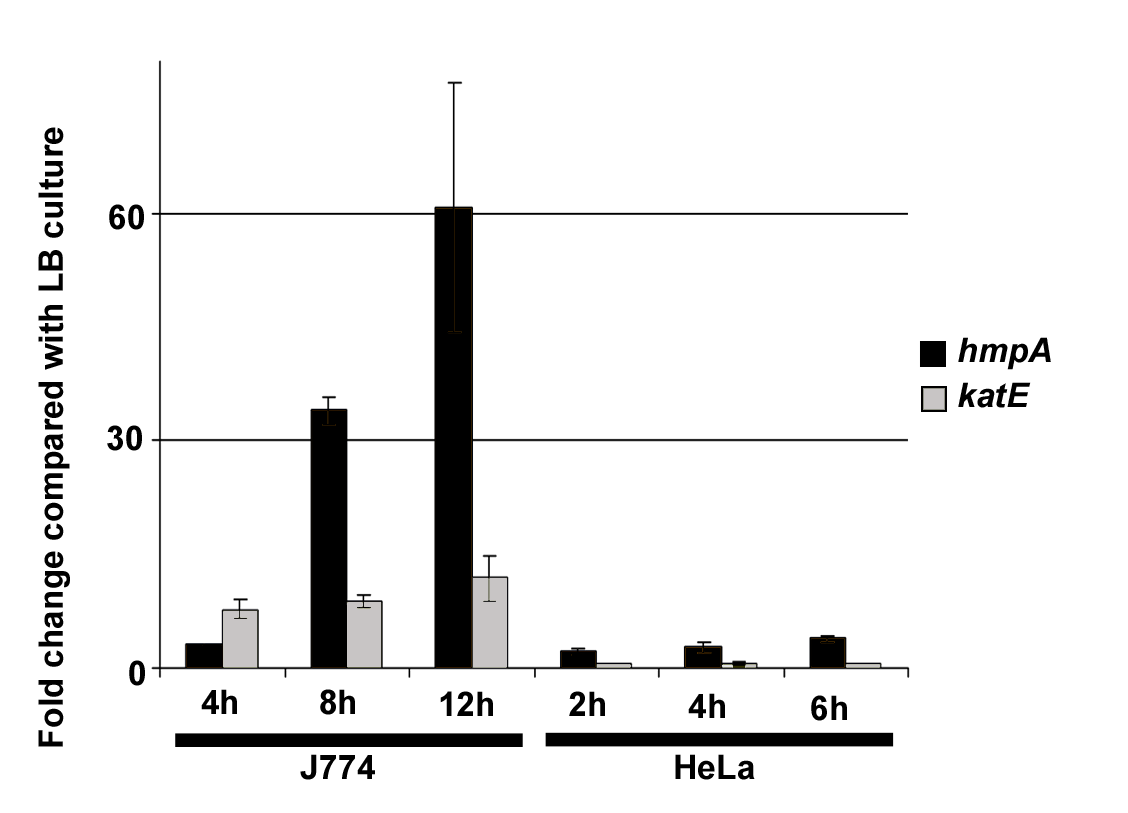

Supplement: Fig. S1 — Salmonella is not exposed to oxidative or nitrosative stressors within epithelial cells. Transcriptomic data are shown for the S. Typhimurium hmpA and katE genes inside both macrophage and epithelial cells. Data for the expression of these genes are taken from Table S1. Error bars indicate the standard error of the mean. [file cmi0010-0958-SD1.tif]
